# Intranasal Administration of *N*-acetyl-L-cysteine Combined with Cell-Penetrating Peptide-Modified Polymer Nanomicelles as a Potential Therapeutic Approach for Amyotrophic Lateral Sclerosis

**DOI:** 10.3390/pharmaceutics14122590

**Published:** 2022-11-24

**Authors:** Takumi Kurano, Takanori Kanazawa, Shingo Iioka, Hiromu Kondo, Yasuhiro Kosuge, Toyofumi Suzuki

**Affiliations:** 1 School of Pharmacy, Nihon University, 7-7-1 Narashinodai, Funabashi 274-8555, Chiba, Japan; 2 School of Pharmaceutical Sciences, University of Shizuoka, 52-1 Yada, Suruga-ku, Shizuoka 422-8526, Shizuoka, Japan

**Keywords:** nose-to-brain, nanocarrier, neurodegeneration, spinal cord, *N*-acetyl-L-cysteine

## Abstract

Intranasal administration is a promising route for direct drug delivery to the brain; its combination with nanocarriers enhances delivery. We have previously shown that intranasal administration combined with PEG-PCL-Tat (a nanocarrier) efficiently delivers drugs to the brain and exhibits excellent therapeutic efficacy against brain diseases. We aimed to clarify whether intranasal administration combined with PEG-PCL-Tat represents a useful drug delivery system (DDS) for amyotrophic lateral sclerosis (ALS) pharmacotherapy. We used *N*-acetyl-L-cysteine (NAC) as a model drug with low transferability to the spinal cord and determined the physicochemical properties of NAC/PEG-PCL-Tat. After intranasal administration of NAC/PEG-PCL-Tat, we measured the survival duration of superoxide dismutase-1 G93A mutant transgenic mice (G93A mice), widely used in ALS studies, and quantitatively analyzed the tissue distribution of NAC/PEG-PCL-Tat in ddY mice. The mean particle size and zeta potential of NAC/PEG-PCL-Tat were 294 nm and + 9.29 mV, respectively. Treatment with repeated intranasal administration of NAC/PEG-PCL-Tat considerably prolonged the median survival of G93A mice by 11.5 days compared with that of untreated G93A mice. Moreover, the highest distribution after a single administration of NAC/PEG-PCL-Tat was measured in the spinal cord. These results suggest that intranasal administration combined with PEG-PCL-Tat might represent a useful DDS for ALS therapeutics.

## 1. Introduction

Amyotrophic lateral sclerosis (ALS) is a neurodegenerative disease characterized by selective degeneration of the upper and lower motor neurons, causing muscle atrophy, which ultimately leads to death due to respiratory failure. There are 2 types of ALS: familial ALS, which represents 10% of cases, and sporadic (idiopathic) ALS, which represents 90% of cases. It has been reported that approximately 20% of familial ALS patients are associated with mutations in the gene encoding copper/zinc superoxide dismutase 1 (SOD1) [1]. Transgenic mice (G93A mice) carrying the mutant human SOD1 gene with the glycine 93 changed to alanine (*SOD1*^G93A^) are widely used as mouse models for mechanistic and therapeutic studies of ALS. Several hypotheses for ALS pathogenesis include the involvement of oxidative stress, mitochondrial dysfunction, excitotoxicity, neuroinflammation, and protein aggregation [2]. Riluzole, a glutamate release inhibitor, and edaravone, a free radical scavenger, were developed for ALS treatment based on these etiological hypotheses. These drugs have been approved for use in treating ALS in several countries despite their limited beneficial effects.

The antioxidant *N*-acetyl-L-cysteine (NAC) has been reported to decrease the levels of reactive oxygen species that are elevated as a product of human neuroblastoma SH-SY5Y expressing *SOD1*^G93A^, a gene predisposing individuals to developing ALS [3]. In addition, an in vitro study of NAC showed that it can considerably protect against neuronal death induced by 4-hydroxy-2-nonenal, the levels of which are increased in the spinal cord of ALS patients and G93A mice [4]. Therefore, NAC is considered a promising ALS drug candidate. However, NAC suffers suboptimal efficacy in in vivo studies. It has been reported that NAC administered in drinking water or subcutaneously to G93A mice at 120 days of age, before the onset of paresis, had no significant therapeutic effect on survival [5]. Thus, a recurring issue is that drugs show remarkable effects in in vitro studies, while failing to produce the same effects in vivo. The primary factor is thought to be that barriers, including the blood–brain barrier (BBB) and blood–cerebrospinal fluid barrier, restrict drug transfer to the brain and spinal cord. As motor neuron degeneration in the spinal cord is the primary pathogenesis of ALS, even if promising therapeutic drugs for ALS are developed, it will be necessary to deliver the drugs not only to the brain but also to deeper areas of the central nervous system, including the lumbar spinal cord. Furthermore, it has been reported that the treatment modality of macromolecules or small water-soluble molecules cannot gain access to the brain and spinal cord due to these barriers [6]. Additionally, NAC, a small water-soluble molecule, cannot gain access to the brain and spinal cord by intravenous or intraperitoneal administration [7]. Therefore, the delivery of NAC to the spinal cord, the primary degenerative area of ALS, via a drug delivery system (DDS) would be expected to improve its effectiveness in treating ALS.

Recently, intranasal administration has received attention as a non-invasive route for drug delivery to the brain [8,9], as intranasal administration can transport drugs directly to the brain from the nasal cavity, bypassing the BBB (nose-to-brain) [10]. Water-soluble compounds such as antibodies, insulin, and dextran administered intranasally have been hypothesized to reach the brain parenchyma and cerebrospinal fluid (CSF) from the nasal cavity along perivascular and/or perineural spaces of the olfactory and trigeminal nerves [11,12,13]. Furthermore, previous studies have shown that biopharmaceuticals such as antibodies and peptides, which do not cross the BBB, are enabled to reach the brain by intranasal administration in rodents [11,14,15], non-human primates [16], and humans [17]. In addition, intranasal administration can avoid hepatic first-pass metabolism and reduce the risk of systemic side effects. Moreover, patients can easily self-administer medication intranasally, which is expected to improve treatment adherence. Recently, various studies have attempted to enhance drug delivery to the brain via nose-to-brain. For example, cell-penetrating peptides (CPP) enhance penetration across the mucosal epithelium. Therefore, their combined use with intranasal administration increases the amount of drugs distributed to the brain, resulting in improved therapeutic effects [18,19,20]. The combination of nanocarriers and intranasal administration has been reported to enhance translocation to the brain, as surface modification of the nanocarriers with specific ligands can achieve targeted delivery and protect the drug from enzymes in the nasal cavity [21,22]. We have previously developed CPP-modified polymer micelles (PEG-PCL-Tat) consisting of a Tat peptide chemically modified on a polyethylene glycol-polycaprolactone (PEG-PCL) block copolymer. We have shown that intranasal administration combination with PEG-PCL-Tat allowed efficient permeation of hydrophilic macromolecules through the nasal mucosa and enhanced the brain distribution [23]. Furthermore, we have successfully delivered drugs and nucleic acids to brain tumor rat models as well as cerebral ischemia-reperfusion injury rat models and demonstrated their therapeutic effects following intranasal administration in combination with PEG-PCL-Tat [24,25,26,27]. Moreover, we have reported that polyethylene glycol (PEG)-modified liposomes with a near neutral charge are the most suitable nanocarriers to deliver drugs to a wide area of the brain and spinal cord [28].

Most nose-to-brain studies utilizing nanocarriers have targeted brain diseases; hence, there have been almost no reports of nose-to-brain studies that examine the therapeutic effects on animal model of spinal cord disease pathophysiology and quantitatively evaluate small water-soluble compounds for delivery from the nose to the spinal cord. In this study, we aimed to clarify whether intranasal administration in combination with our nanocarrier represents a useful DDS for ALS pharmacotherapy using G93A mice, which show pathological conditions and symptoms similar to those of ALS patients [29]. Specifically, NAC was selected as the model compound, as it has low transferability to the brain and spinal cord [7]. The physicochemical properties of NAC/PEG-PCL-Tat were evaluated and the effects of daily intranasal administration of NAC/PEG-PCL-Tat on the survival duration of G93A mice were examined. In addition, we quantitatively evaluated drug distribution from the nose to the spinal cord following a single intranasal administration of NAC/PEG-PCL-Tat in healthy mice.

## 2. Materials and Methods

### 2.1. Radioisotopes and Chemicals

Methoxy poly(ethylene glycol)-block-poly(ε-caprolactone) (2k-2k; PEG-PCL block copolymer) was purchased from Sigma-Aldrich Co. (Milwaukee, WI, USA), and Tat peptide (GRKKRRQRRRG) was purchased from BEX Co., Ltd. (Tokyo, Japan). NAC was purchased from Sigma-Aldrich Co. [Cystein-1-^14^C] *N*-Acetyl-L-cysteine ([^14^C]-NAC, specific activity: 55 mCi/mmol, purity: >98%) was purchased from American Radiolabeled Chemicals Inc. (St. Louis, MO, USA). All other reagents used in this study were commercial products without further purification.

### 2.2. Synthesis of PEG-PCL-Tat Micelles

The PEG-PCL-Tat was synthesized as previously described [27,30]. Briefly, the Tat peptide (0.02 mmol) and PEG-PCL block copolymer (0.02 mmol) were dissolved in dimethylformamide. Water-soluble carbodiimide hydrochloride (0.02 mmol) and 4-dimethylaminopyridine (0.02 mmol) were then added to this solution. To form an ester bond between the Gly-COOH on the C terminus of Tat peptide and the –OH group on PEG-PCL, the solution was stirred at room temperature (25 °C) for 24 h. The reaction solution was transferred to a dialysis membrane (Molecular weight cut-off: 3.5 kDa) suitable for use with organic solvents and dialyzed against ultrapure water for 24 h under continuous stirring (on a magnetic stirrer) at 150 rpm. Subsequently, the reaction solution was freeze-dried to obtain PEG-PCL-Tat.

### 2.3. Preparation of the NAC Containing PEG-PCL-Tat Micelles Solution

The PEG-PEL-Tat solution with a concentration of 5 mg/mL or 25 mg/mL was prepared by completely dissolving through pipetting 1.5 or 7.5 mg of PEG-PCL-Tat in 300 µL of 10 mM 2-[4-(2-hydroxyethyl)-1-piperazinyl] ethanesulfonic acid (HEPES) buffer (pH 7.4), respectively. NAC was dissolved in 10 mM HEPES buffer (pH 7.4) to prepare a stock solution at a concentration of 100 mg/mL. The NAC solution (20 or 100 mg/mL) was mixed by adding, in equal volumes, the PEG-PCL-Tat solution (5 or 25 mg/mL). The resulting solution was incubated at room temperature (25 °C) for 30 min to obtain 0.2 mg NAC/PEG-PCL-Tat (0.2NAC/PEG-PCL-Tat) or 1 mg NAC/PEG-PCL-Tat (NAC/PEG-PCL-Tat) as a concentration of 10 mg/mL or 50 mg/mL of NAC solution. In 0.2NAC/PEG-PCL-Tat and NAC/PEG-PCL-Tat solutions, the weight ratio of PEG-PCL-Tat (1.5 or 7.5 mg) to NAC (6 or 30 mg) was 1:4. The PEG-PCL-Tat solution (12.5 mg/mL) without NAC was prepared by diluting the PEG-PCL-Tat solution (25 mg/mL) in 10 mM HEPES buffer (pH 7.4). The NAC containing PEG-PCL-Tat solution was used in the experiment immediately after the preparation.

### 2.4. Physicochemical Characterization of the NAC Containing PEG-PCL-Tat Micelles Solution

The mean particle size, polydispersity index (PDI), and zeta potential of the PEG-PCL-Tat, 0.2NAC/PEG-PCL-Tat, and NAC/PEG-PCL-Tat solutions were measured after diluting them to an appropriate concentration range via the Zetasizer Ultra (Malvern Instruments, Worcestershire, UK).

### 2.5. Animals

All efforts were made to minimize the number of animals used and their distress. Animal experiments were carried out after obtaining approval from the Nihon University Animal Care and Use Committee (Approval no.: AP19PHA026-1 and AP20PHA006-1) (Tokyo, Japan). Animals were housed under controlled conditions (temperature 23 ± 1 °C, relative humidity 50 ± 10%, and 12 h light/dark cycles [light on 8:00 a.m. to 8:00 p.m.]) and with ad libitum access to feed and water.

#### 2.5.1. G93A Mice

Transgenic mice of the B6SJL-TgN (SOD1-G93A) 1Gur strain were used as an ALS mouse model, purchased from the Jackson Laboratory (Bar Harbor, ME, USA). The hemizygous G93A mice were obtained by mating male G93A mice with wild-type (WT) females. Mice were genotyped by polymerase chain reaction (PCR) using genomic DNA isolated from blood as previously described [31]. Only male mice weighing 25–30 g were used in this study to control for possible gender differences.

#### 2.5.2. ddY Mice

Four-week-old ddY mice (male) were obtained from Japan SLC, Inc. (Shizuoka, Japan) for the pharmacokinetic investigation of the intranasal administration. Mice weighing 25–35 g were used for the experiments after being pre-fed as previously reported [28].

### 2.6. Intranasal Administration

Intranasal administration was performed as previously described [32,33]. In brief, the nasal area of mice under isoflurane (4% induction, 2% maintenance) inhalation anesthesia was covered with an openable inhalation mask (SN-487-70-09, Shinano Seisakusho, Tokyo, Japan). After opening the silicone cap of the mask, mice were administered a total volume of 20 µL dosing solution at 30-s intervals over 10 min, via 1-µL doses alternatively into each naris (1 µL/30 s). The dosing solution was administered by timing the droplet from the tip of the microtip to be brought close to the nasal cavity and synchronized with the respiration of the mouse, thereby allowing for spontaneous aspiration. Intranasal administration of 0.2NAC/PEG-PCL-Tat or NAC/PEG-PCL-Tat solutions was performed in a total volume of 20 µL per mouse, equivalent to receiving 0.2 mg or 1.0 mg of NAC, respectively.

### 2.7. Drug Administration and Survival Analyses

For survival analyses, a total of 108 male G93A mice were randomly assigned into six groups as shown in Table 1. Starting at 105 days of age, which is shortly after the onset of ALS [34], G93A mice were treated with NAC or NAC complex (NAC/PEG-PCL-Tat) on weekdays (5 d a week). In cases when the 105th day fell on a holiday or national holiday in Japan, treatments were started on day 106 or 107. The treatment was continued until the endpoint. The endpoint was defined as the date on which a mouse could not right itself within 30 s after being placed on its side [35]. At that point, the G93A mice were euthanized with CO_2_. The median survival duration is the time period taken for 50% of G93A mice to die. The mean survival time is the average amount of days that G93A mice reached the endpoint. In the group that received intranasal administration (Groups 3–6) of Table 1, some mice did not show spontaneous respiration during administration or until awakening from anesthesia. Accordingly, we excluded these mice from the assessment of survival duration.

### 2.8. Pharmacokinetics Following Intranasal Administration of [^14^C]-NAC/PEG-PCL-Tat Micelles in ddY Mice

#### 2.8.1. Preparation of [^14^C]-NAC/PEG-PCL-Tat

NAC was dissolved in 10 mM HEPES buffer (pH 7.4) to prepare the stock solution (200 mg/mL). For radioactive tracer experiments, the [^14^C]-NAC solution, which contained unlabeled-NAC equivalent to that of group 3, was prepared by adding an equal volume of 10 mM HEPES buffer (pH 7.4) into the NAC solution (25 µL). The [^14^C]-NAC/PEG-PCL-Tat solution (10 µCi/mL), containing 1 mg NAC, was prepared by adding the NAC solution as described above dropwise into the PEG-PCL-Tat solution and further mixed and incubated at room temperature (25 °C) for 30 min. Mice were administered either 20 µL of [^14^C]-NAC or [^14^C]-NAC/PEG-PCL-Tat solution, each containing 1 mg of NAC.

#### 2.8.2. Blood Sampling after the Intranasal Administration

Blood samples (50 µL) were collected from the tail vein of ddY mice at 0, 3, 15, 30, 60, and 90 min after an intranasal administration of [^14^C]-NAC or [^14^C]-NAC/PEG-PCL-Tat solution, each containing unlabeled-NAC (1 mg). Plasma samples (20 µL) were obtained through centrifugation (15 min, 2100× *g*).

### 2.9. Collection of CSF, Plasma, and Tissues Following an Intranasal Administration to ddY Mice

CSF and tissue samples were obtained at 60 min after an intranasal administration of [^14^C]-NAC or [^14^C]-NAC/PEG-PCL-Tat solution to ddY mice. CSF was slowly withdrawn (approximately 20 µL) from the cisterna magna through a needle (30 G) connected to a cannula. Plasma samples (approximately 0.5 mL) were obtained through blood centrifugation (15 min, 2100× *g*), collected from the heart. Tissues (trigeminal nerve, brain, spinal cord [including the thoracic spinal cord and lumbar spinal cord], lungs, liver, and kidneys) were obtained after systemic perfusion with phosphate-buffered saline (pH 7.4) using a peristaltic pump until the circulating fluid was decolored. Subsequently, each tissue sample was weighed (wet weight) and prepared as previously described [33].

### 2.10. Determination of [^14^C] Radioactivity

The radioactivity of [^14^C] in plasma, CSF, or tissues was measured using a liquid scintillation counter (Tri-Carb 4810TR, PerkinElmer Inc., Waltham, MA, USA), as per our previous report [33]. The percentage of injected dose per gram of tissue (%ID/g tissue) or per milliliter of plasma or CSF (%ID/mL plasma or CSF) was calculated for each sample. A maximum drug concentration (C_max_) was determined as the highest value among the calculated values of %ID/mL plasma. The area under the plasma concentration–time curve (AUC_0–90_) was calculated by the linear trapezoidal method using the [^14^C]-NAC radioactivity in the plasma to the last time point (*t* = 90 min).

### 2.11. Statistical Analysis

Statistical analyses were performed using GraphPad Prism 9 (GraphPad Software Inc., San Diego, CA, USA). Data were expressed as the mean ± standard error (SE) or standard deviation (SD). Student’s *t*-test was used for comparisons between two groups. The significance of differences between multiple groups was analyzed using a one-way analysis of variance (ANOVA) followed by Tukey’s post hoc test. Comparisons of survival duration analyzed by the Kaplan–Meier method were performed using the log-rank test. The differences were considered to be significant at *p* < 0.05.

## 3. Results

### 3.1. Physicochemical Characterization of NAC/PEG-PCL-Tat Micelles

The physicochemical characterization of PEG-PCL-Tat, 0.2NAC/PEG-PCL-Tat, and NAC/PEG-PCL-Tat is demonstrated in Table 2. The mean particle size and PDI values of 0.2NAC/PEG-PCL-Tat and NAC/PEG-PCL-Tat showed no significant difference compared with those of PEG-PCL-Tat. In contrast, the zeta potential of NAC/PEG-PCL-Tat was significantly decreased compared with that of PEG-PCL-Tat and 0.2NAC/PEG-PCL-Tat (*p* = 0.00001 and *p* = 0.00009, respectively). The results revealed that the zeta potential decreases in a NAC concentration-dependent manner. It has been reported that nanoparticles with zeta potentials of −10 mV to +10 mV are generally regarded as neutrally charged, while nanoparticles with an absolute value of zeta potentials exceeding ± 10 mV are positively or negatively charged [36]. PEG-PCL-Tat and 0.2 NAC/PEG-PCL-Tat exhibited positive charges, while NAC/PEG-PCL-Tat exhibited neutral charges.

### 3.2. Effect of NAC on Survival Duration of G93A Mice

**Figure 1:**
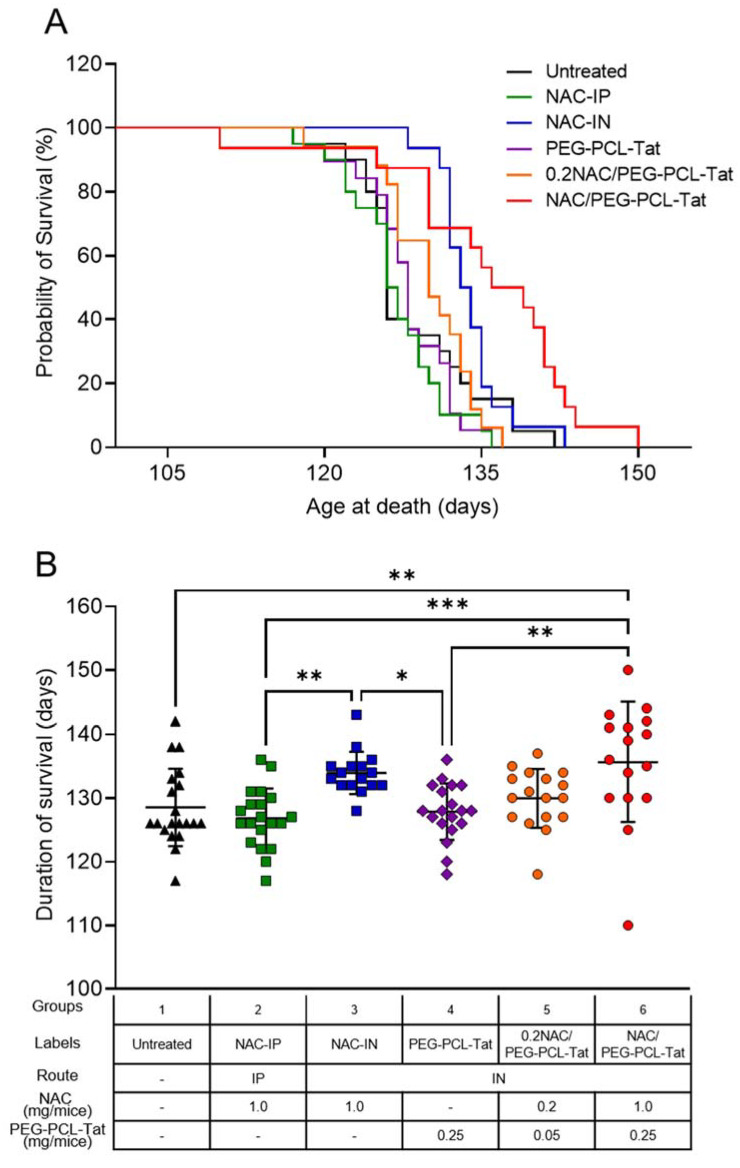
Lifespan of G93A mice treated with intranasal administration of NAC/PEG-PCL-Tat. G93A mice were treated with NAC-IP (1 mg), NAC-IN (1 mg), PEG-PCL-Tat (IN), 0.2NAC/PEG-PCL-Tat (IN; 0.2 mg), or NAC/PEG-PCL-Tat (IN; 1 mg), starting at a late symptomatic stage (15 weeks old). (**A**) Survival curves were analyzed using Kaplan–Meier survival analysis with the log-rank test; (**B**) The graph shows the lifespan comparative result. The values are presented as mean ± SD. Statistical significance was determined using one-way ANOVA followed by Tukey’s post hoc test. * *p* < 0.05, ** *p* < 0.01, and *** *p* < 0.001.

We examined the effect of NAC on the survival of G93A mice. The treatment with NAC-IP (1.0 mg), NAC-IN (1.0 mg), PEG-PCL-Tat, 0.2NAC/PEG-PCL-Tat (0.2 mg), or NAC/PEG-PCL-Tat (1 mg) was started in 15-week-old mice (an early symptomatic stage). As shown in Figure 1A, the median survival duration of untreated mice, NAC-IP–treated mice, PEG-PCL-Tat–treated mice, and 0.2NAC/PEG-PCL-Tat–treated mice was 126.0, 126.5, 128.0, and 130.0 d, respectively, and there was no significant difference between groups. Moreover, the median survival duration of NAC-IN (1.0 mg)–treated mice was 133.5 d, showing no significant difference compared with that of untreated mice. Similarly, there was no difference in the median survival duration between untreated mice and NAC-IP (1.0 mg)–treated mice. Despite the non-significant improvement in survival observed in 0.2NAC/PEG-PCL-Tat (0.2 mg)–treated mice, the NAC/PEG-PCL-Tat (1.0 mg) treatment extended median survival duration by 11.5 d, from 126.0 (untreated mice) to 137.5 d (*p* = 0.0345; Figure 1A). These results revealed that intranasal administration of NAC with PEG-PCL-Tat as a nanocarrier increased the survival duration of G93A mice in a concentration-dependent manner.

The mean survival time of G93A mice is shown in Figure 1B. Similar to the result illustrated in Figure 1A, the mean survival time of untreated mice, NAC-IP–treated mice, PEG-PCL-Tat–treated mice, and 0.2NAC/PEG-PCL-Tat–treated mice was 128.5, 126.8, 127.8, and 129.9 d, respectively. There were no significant differences between groups. In contrast, NAC/PEG-PCL-Tat (1.0 mg) treatment significantly extended the mean survival duration of G93A mice when compared with that of untreated mice (135.6 d vs. 128.5 d, *p* = 0.0042; Figure 1B). There was no difference between the mean survival time of NAC-IN (1.0 mg)–treated mice and that of untreated mice (133.9 d vs. 128.5 d). NAC/PEG-PCL-Tat–treated mice were the only group that markedly extended mean survival time compared with that observed in untreated mice, thus indicating that intranasal administration combined with PEG-PCL-Tat is the most efficacious for the NAC therapy.

### 3.3. Plasma Time-Dependent Concentration of [^14^C]-NAC/PEG-PCL-Tat Micelles after a Single Intranasal Administration

**Figure 2:**
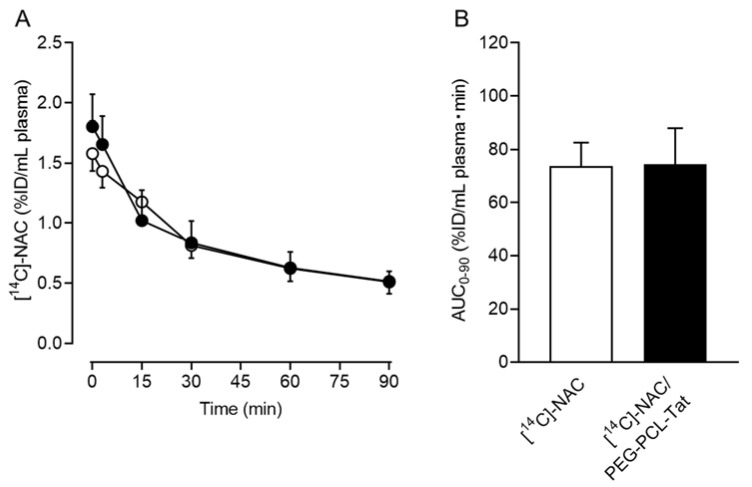
Concentration-time profiles of [^14^C]-NAC in plasma after a single intranasal administration to ddY mice and the area under the plasma concentration time curve (AUC). (**A**) Plasma was obtained from the blood collected at the designated time after the intranasal administration of [^14^C]-NAC (○) or [^14^C]-NAC/PEG-PCL-Tat (●). The %ID in mL plasma represents the ratio of the distribution in plasma to the dosing volume of an intranasally administered drug. Values represent the mean ± SE (*n* = 6 or 7); (**B**) AUC_0–90_ was calculated using a linear trapezoidal method and values for plasma concentration to the last time point (*t* = 90 min) were obtained. Values represent the mean ± SE (*n* = 6 or 7). The significant differences in mean plasma concentrations at the same time or AUC_0–90_ between the two groups were analyzed using a *t*-test.

Figure 2A shows the plasma time-dependent concentration after a single intranasal administration of [^14^C]-NAC/PEG-PCL-Tat to ddY mice. The post-administration C_max_ of [^14^C]-NAC/PEG-PCL-Tat was 1.81 ± 0.7% ID/mL plasma, showing a trend slightly higher than that measured following the administration of [^14^C]-NAC without PEG-PCL-Tat. In the [^14^C]-NAC/PEG-PCL-Tat group, the concentration in plasma trended higher than that measured in the [^14^C]-NAC group at early time points after the intranasal administration (at 10 and 13 min from administration commencement). In the elimination phase, 30 to 90 min after the intranasal administration (40 to 100 min after the start of the administration), the plasma concentrations of [^14^C]-NAC/PEG-PCL-Tat group showed no difference in concentration compared with that of [^14^C]-NAC group and both plasma concentrations decreased at a similar pace over time (Figure 2A). However, there were no significant differences between groups at either point. AUC_0–90_ was calculated using a linear trapezoidal method with the values for plasma concentration until the last time point (*t* = 90 min). The AUC_0–90_ in the [^14^C]-NAC/PEG-PCL-Tat group was approximately 75% ID/mL plasma min and was not significantly different from that of the [^14^C]-NAC group (Figure 2B).

### 3.4. Drug Distribution in Tissue after a Single Intranasal Administration of [^14^C]-NAC/PEG-PCL-Tat Micelles

**Figure 3:**
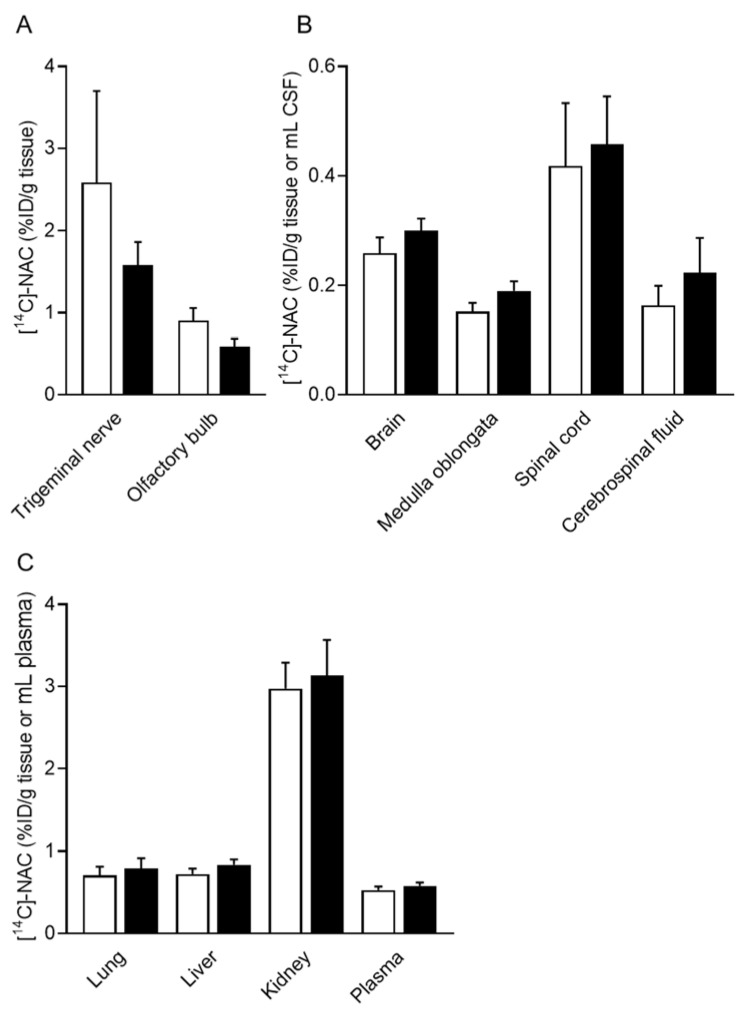
Tissue distribution of [^14^C]-NAC after a single intranasal administration to ddY mice. Each tissue, CSF, or plasma sample was collected 60 min after the intranasal administration of [^14^C]-NAC or [^14^C]-NAC/PEG-PCL-Tat. (**A**–**C**) The opened and closed columns represent [^14^C]-NAC and [^14^C]-NAC/PEG-PCL-Tat, respectively. Each column presents the mean ± SE (*n* = 10 or 11). Significance was assessed using a *t*-test.

Figure 3 shows the drug distribution in the tissue 60 min after the intranasal administration of [^14^C]-NAC/PEG-PCL-Tat. In the [^14^C]-NAC/PEG-PCL-Tat group, the [^14^C]-NAC distribution to the trigeminal nerve or olfactory bulb was approximately 40% lower than that in the [^14^C]-NAC without PEG-PCL-Tat group (Figure 3A). For central regions such as the brain, medulla oblongata, spinal cord, and CSF, the distribution of [^14^C]-NAC trended higher in the [^14^C]-NAC/PEG-PCL-Tat group than in the [^14^C]-NAC without PEG-PCL-Tat group (Figure 3B). In CSF, the [^14^C]-NAC distribution after an intranasal administration of [^14^C]-NAC/PEG-PCL-Tat was 1.4-fold higher than that measured in the [^14^C]-NAC without PEG-PCL-Tat group. For the spinal cord, the distribution of [^14^C]-NAC/PEG-PCL-Tat was increased compared with that of [^14^C]-NAC 60 min after the intranasal administration and was the highest among the other tissue (0.46 ± 0.09% ID/g tissue). In the [^14^C]-NAC/PEG-PCL-Tat group, the [^14^C]-NAC amount distributed within the spinal cord was 1.5- and 2.1-fold higher than that measured in the brain and CSF, respectively (Figure 3B). However, there were no significant differences in the amount of [^14^C]-NAC distributed in any tissue or CSF of the central regions. Similarly, there was no significant difference in the amount of [^14^C]-NAC distributed in the peripheral tissues analyzed (Figure 3C). Thus, no significant differences were observed between the two groups in any of the tissues in the single intranasal administration.

## 4. Discussion

This study aimed to establish a non-invasive and efficient DDS for brain and spinal cord delivery of therapeutics. We selected NAC as a model compound with low transferability to the brain and spinal cord, and investigated the effect of NAC by intranasal delivery, which has been shown to bypass the BBB and blood–cerebrospinal fluid barrier. Furthermore, we combined NAC with PEG-PCL-Tat, which enhances nasal mucosal permeability and brain distribution. We assessed the delivery effectiveness using survival duration of G93A mice. In addition, we quantitatively investigated the distribution of NAC in the brain and spinal cord of healthy mice.

NAC/PEG-PCL-Tat was prepared by mixing an equal volume of NAC and PEG-PCL-Tat micelles in a weight ratio of 1:4. NAC is a water-soluble compound and thus cannot be encapsulated in the micelle, PEG-PCL-Tat. Previous studies showed that positively charged Tat peptides are able to form complexes with negatively charged plasmid DNA and siRNA via electrostatic interaction [27,37]. NAC at near neutral pH is negatively charged [38]. In this study, NAC was dissolved in HEPES buffer at a pH of 7.4, suggesting that electrostatic interactions occurred between positively charged Tat peptide and the negatively charged NAC. Therefore, part of the NAC has been loaded in the surface of PEG-PCL-Tat, which may have caused a NAC concentration-dependent decrease in zeta potential due to charge neutralization (Table 2). Particle sizes of nanocarriers utilized in nose-to-brain delivery have been reported in the 50–500 nm range [39]. In addition, the PDI value of NAC/PEG-PCL-Tat was slightly above 0.5. However, we considered it to be close to the usually recommended value (<0.5) [40]. We have previously reported that PEG-modified neutral liposomes, among PEG-modified positively, neutrally, and negatively charged liposomes, showed higher distribution in a wide area of the brain and spinal cord via the olfactory and trigeminal nerve pathways [28]. Therefore, NAC/PEG-PCL-Tat is expected to enhance the nose-to-brain delivery due to its physicochemical characterization.

Next, we examined the effect of intranasally administered NAC/PEG-PCL-Tat on the survival duration of G93A mice. The intraperitoneal administration of NAC alone as well as intranasal administration of NAC and PEG-PCL-Tat alone were also examined. No significant differences were observed between the survival duration of NAC-IP– or NAC-IN–treated mice when compared with the untreated mice (Figure 1). These results suggest that changing the administration route of NAC to an intranasal route was not sufficient to provide a therapeutic effect. Similarly, no significant difference was observed in the PEG-PCL-Tat–treated mice compared with the untreated mice, indicating that the PEG-PCL-Tat alone did not provide a therapeutic effect. However, the intranasal administration of NAC with PEG-PCL-Tat as a nanocarrier increased the survival duration of G93A mice in a concentration dependent manner, with the median and mean survival of the NAC/PEG-PCL-Tat–treated mice being markedly increased by 11.5 d (9.1%) and 7.1 d (5.5%), respectively, when compared with those of the untreated mice (Figure 1). A previous study suggested that NAC administered in drinking water to G93A mice 4–5 weeks of age (before onset phase) prolonged their mean survival time by 8.6 d (6.7%) [41]. Therefore, this study revealed that the therapeutic effect of intranasal administration of NAC/PEG-PCL-Tat is comparable to that of preventative treatment even after ALS onset. In addition, despite riluzole and edaravone currently being approved by the Food and Drug Administration (FDA) and in Japan for the treatment of ALS, it has been reported that neither riluzole administered in drinking water, nor intraperitoneal administration of edaravone to G93A mice after ALS onset prolonged their survival [42,43]. These results suggested that the intranasal administration of NAC/PEG-PCL-Tat shows a therapeutic effect that outperforms the in vivo results from studies investigating riluzole administered in drinking water and the intraperitoneal administration of edaravone to G93A mice. As no valid early diagnostic methods have yet been established for sporadic ALS, most drug treatments are initiated after the onset of motor dysfunction or other symptoms. Our results suggest that intranasal administration of PEG-PCL-Tat could be a very useful drug delivery strategy for the treatment of ALS, even after disease onset.

Next, we analyzed the pharmacokinetics of a single intranasal administration of [^14^C]-NAC/PEG-PCL-Tat to ddY mice, as the survival duration of the NAC/PEG-PCL-Tat–treated mice was considerably extended compared with that of the untreated mice. The plasma time-dependent concentration indicates that the interaction between NAC and PEG-PCL-Tat is maintained at early time points after intranasal administration, whereas this interaction is lost after 30 min (Figure 2A).

Furthermore, we quantitatively analyzed drug distribution in the brain, medulla oblongata, spinal cord, and CSF after a single intranasal administration of [^14^C]-NAC/PEG-PCL-Tat. In the trigeminal nerve and olfactory bulb, the [^14^C]-NAC distribution in the [^14^C]-NAC/PEG-PCL-Tat group was lower than in the [^14^C]-NAC group (Figure 3A). Similar to the transfer to the blood following intranasal administration of [^14^C]-NAC/PEG-PCL-Tat, the transfer of [^14^C]-NAC/PEG-PCL-Tat into the trigeminal and olfactory nerve pathways from the nasal cavity is expected to possess a similar time point. This suggests that the reason for the lower [^14^C]-NAC distribution in the trigeminal nerve and olfactory bulb observed in the [^14^C]-NAC/PEG-PCL-Tat group is that [^14^C]-NAC/PEG-PCL-Tat had already reached the brain and medulla oblongata via the trigeminal nerve and olfactory bulb 60 min after administration. This is supported by our previous report that PEG-PCL-Tat is transferred to the brain via the olfactory and trigeminal nerve pathways because, after intranasal administration of Alexa-dextran/PEG-PCL-Tat, the fluorescence of Alexa-dextran was observed in the olfactory bulb at 15 min and then in the olfactory bulb and brainstem at 60 min [23]. The highest improvement in the relative distribution ratio of the [^14^C]-NAC/PEG-PCL-Tat group when compared with that in the [^14^C]-NAC group was in the CSF (Figure 3B). The [^14^C]-NAC in CSF is thought to flow into the brain via the glymphatic system [44], a circulatory mechanism between CSF and interstitial fluid in perivascular space (PVS). It has been previously shown that the PVS is involved in some portion of drug diffusion through the brain, as fluorescence was observed in perivascular and the PVS of cerebral arteries after intranasal administration of fluorescently labeled dextran [13]. Conversely, concerning the pathway from the brain to the spinal cord, it is unclear how diffusion in the brain affects distribution to the spinal cord. However, it has been reported that PVS was observed around arterioles and venules as well as throughout the spinal cord white and grey matter, while the tracer injected into the cisterna magna appeared to be spreading from the PVS into the surrounding parenchyma [45]. Thus, the [^14^C]-NAC in the CSF is speculated to flow similarly into the spinal cord via the glymphatic system. We showed that the increased distribution to the brain, medulla oblongata, and spinal cord in the [^14^C]-NAC/PEG-PCL-Tat group was accompanied by an increased distribution to the CSF when compared with that observed in the [^14^C]-NAC group (Figure 3B). Although no significant difference was observed between the two groups in all regions after a single administration, the [^14^C]-NAC/PEG-PCL-Tat group tended to exhibit increased [^14^C]-NAC distribution in each tissue when compared with that measured in the [^14^C]-NAC group, thereby suggesting that repeated administration might have extended survival.

## 5. Conclusions

In this study, we demonstrated that intranasal administration of NAC, which has low transferability from the blood to the brain and spinal cord, extensively extended the survival of G93A mice (an ALS animal model) when combined with a PEG-PCL-Tat nanocarrier. After a single intranasal administration to healthy ddY mice, the [^14^C]-NAC distribution to the brain and spinal cord of [^14^C]-NAC/PEG-PCL-Tat group slightly increased compared with that observed in [^14^C]-NAC without PEG-PCL-Tat group. We showed that intranasal administration combined with PEG-PCL-Tat is a useful DDS not only for the treatment of brain diseases such as brain tumors and cerebral ischemia-reperfusion injury but also for ALS with the primary pathophysiology of the spinal cord. Future optimizations of the nanocarrier are expected to provide wider applicability.

## Figures and Tables

**Table 1:** Grouping and treatments of G93A mice.

Groups	Labels	Route of Administration	Dosing Volume (µL)	NAC (mg/Mice)	PEG-PCL-Tat (mg/Mice)	Number of Animals
1	Untreated	-	-	-	-	20
2	NAC-IP	IP	100	1.0	-	20
3	NAC-IN	IN	20	1.0	-	16
4	PEG-PCL-Tat	IN	20	-	0.25	19
5	0.2NAC/PEG-PCL-Tat	IN	20	0.2	0.05	17
6	NAC/PEG-PCL-Tat	IN	20	1.0	0.25	16

IP, intraperitoneal; IN, intranasal; NAC, *N*-Acetyl-L-cysteine; PEG-PCL, methoxy poly(ethylene glycol)-block-poly(ε-caprolactone).

**Table 2:** Particle diameter, PDI, and zeta potential of PEG-PCL-Tat, 0.2NAC/PEG-PCL-Tat, and NAC/PEG-PCL-Tat.

Nanocarrier	Mean Particle Size (nm)	PDI	Zeta Potential (mV)
PEG-PCL-Tat	285 ± 6.1	0.496 ± 0.016	+14.1 ± 0.12
0.2NAC/PEG-PCL-Tat	270 ± 8.7	0.612 ± 0.104	+12.9 ± 0.45
NAC/PEG-PCL-Tat	294 ± 7.2	0.541 ± 0.106	+9.29 ± 0.52 ****

The mean particle size, PDI, and zeta potential of PEG-PCL-Tat, 0.2NAC/PEG-PCL-Tat, and NAC/PEG-PCL-Tat were measured three times using a Zetasizer Ultra. Data are expressed as mean ± SD (*n* = 3). Statistical significance was determined using one-way ANOVA followed by Tukey’s post hoc test. **** *p* < 0.0001 in comparison with PEG-PCL-Tat or 0.2NAC/PEG-PCL-Tat.

## Data Availability

Not applicable.
